# Permeability of Gemcitabine and PBPK Modeling to Assess Oral Administration

**DOI:** 10.3390/cimb43030153

**Published:** 2021-12-07

**Authors:** Abigail Ferreira, Rui Lapa, Nuno Vale

**Affiliations:** 1 OncoPharma Research Group, Center for Health Technology and Services Research (CINTESIS), Rua Doutor Plácido da Costa, 4200-450 Porto, Portugal; abigail.ferreira@fc.up.pt; 2 LAQV/REQUIMTE, Laboratory of Applied Chemistry, Department of Chemical Sciences, Faculty of Pharmacy, University of Porto, Rua de Jorge Viterbo Ferreira 228, 4050-313 Porto, Portugal; ruilapa@ff.up.pt; 3 Faculty of Medicine, University of Porto, Alameda Professor Hernâni Monteiro, 4200-319 Porto, Portugal

**Keywords:** gemcitabine, cancer therapy, in silico study, PBPK modeling, GastroPlus™

## Abstract

Gemcitabine is a nucleoside analog effective against several solid tumors. Standard treatment consists of an intravenous infusion over 30 min. This is an invasive, uncomfortable and often painful method, involving recurring visits to the hospital and costs associated with medical staff and equipment. Gemcitabine’s activity is significantly limited by numerous factors, including metabolic inactivation, rapid systemic clearance of gemcitabine and transporter deficiency-associated resistance. As such, there have been research efforts to improve gemcitabine-based therapy efficacy, as well as strategies to enhance its oral bioavailability. In this work, gemcitabine in vitro and clinical data were analyzed and in silico tools were used to study the pharmacokinetics of gemcitabine after oral administration following different regimens. Several physiologically based pharmacokinetic (PBPK) models were developed using simulation software GastroPlus™, predicting the PK parameters and plasma concentration–time profiles. The integrative biomedical data analyses presented here are promising, with some regimens of oral administration reaching higher AUC in comparison to the traditional IV infusion, supporting this route of administration as a viable alternative to IV infusions. This study further contributes to personalized health care based on potential new formulations for oral administration of gemcitabine, as well nanotechnology-based drug delivery systems.

## 1. Introduction

Cancer is one of the most prevalent and mortal diseases and still has an increasing incidence rate. Globally, about one in six deaths is due to cancer [1]. Treatment options include chemotherapy, radiotherapy and/or surgery. Although surgical removal and/or radiotherapy is typically the first recommendation for well-defined solid tumors with a promising prognosis, chemotherapy is administered to almost all cancer patients, even if as an adjuvant treatment. There has been continuous research to improve overall treatment efficacy and reduce associated adverse side effects, as there are several shortcomings in the currently available treatments.

Gemcitabine (2′,2′-difluoro-2′-deoxycytidine or dFdC) is a nucleoside analog and pyrimidine antimetabolite with proven efficacy against a variety of solid tumors, being the first-line treatment for pancreatic cancer and also used in the therapy of ovarian, breast and non-small-cell lung cancer [2,3]. Combinations of gemcitabine with other anticancer agents, such as paclitaxel and platinum analogs oxaliplatin, carboplatin and cisplatin, are also employed [4,5,6]. However, multiple factors limit the efficacy of gemcitabine-based treatments. Gemcitabine is rapidly inactivated in the serum through metabolic deamination by cytidine deaminase (CDA). Additionally, this drug’s binding to plasma proteins is negligible (<10%, [7]), leaving the majority of circulating gemcitabine unbound and available for metabolic modification and inactivation. Gemcitabine is thus very rapidly cleared from the body, having a short half-life (8–17 min). Another drawback limiting the efficacy of this drug is the resistance related to nucleoside transporter deficiency. The human equilibrative nucleoside transporter 1 (hENT1) is primarily responsible for the cellular uptake of gemcitabine, but an underexpression of this transporter is developed in some tumor cells after initial tumor regression [3]. Thus, much higher doses may be required to reach an effective plasma concentration.

Currently, standard treatment with gemcitabine is an intravenous (IV) infusion of 1000 or 1250 mg/m^2^ over 30 min once a week and then follows different treatment cycle schedules [7]. This is an invasive and very uncomfortable method. As previously mentioned, numerous strategies have been developed to improve treatment efficacy and reduce side effects, including the study of drug combinations, chemical modifications and the development of prodrugs. Some strategies aim at surpassing some of the unfavorable physicochemical properties of gemcitabine and improving this drug’s oral bioavailability, to avoid IV administration.

The oral route of administration presents some limitations, the most impactful being the first-pass effect, which significantly diminishes the amount of drug that reaches systemic circulation (in the case of gemcitabine, CDA is present in high levels in the liver and metabolizes gemcitabine to the inactive metabolite 2′,2′-difluorodeoxyuridine, dFdU). Nevertheless, this is undoubtedly a much more convenient and comfortable form of administering drugs. We were encouraged by promising previous results reported for various prodrugs of gemcitabine developed to enhance oral bioavailability [8,9,10], including some studies carried out by our research group regarding gemcitabine conjugates with cell-penetrating peptides (CPPs) [11,12,13].

In this work, the oral route of administration was preliminarily assessed as an alternative to IV infusions, and the pharmacokinetics of gemcitabine after oral administration via a tablet following different treatment regimens with varying doses and dosing intervals were studied and compared to the IV form. Since the single layer of epithelial cells covering the inner intestinal wall is the rate-limiting barrier to the absorption of dissolved compounds administered orally, the permeability of gemcitabine through a monolayer of Caco-2 cells was also evaluated, using a method recognized by the American Food and Drug Administration (FDA) [14]. Several physiologically based pharmacokinetic (PBPK) models were then developed, and the main PK parameters were predicted using simulation software GastroPlus™. This software integrates an advanced compartmental absorption and transit model (ACAT) and uses a set of differential equations to model the amount of drug that is released, dissolved and absorbed for all physiologically predefined compartments. The plasma concentration–time profiles and the regional absorption throughout the different compartments of the gastrointestinal tract were also evaluated and are presented here.

## 2. Materials and Methods

### 2.1. Cell Culture

Human colon adenocarcinoma cells, from cell line Caco-2 (passage 25–47, kindly provided by the Department of Biomedicine of the Faculty of Medicine of University of Porto (Professor Fátima Martel), and previously acquired via ATCC) were routinely maintained in Dulbecco’s modified Eagle’s medium (DMEM), supplemented with 10% fetal bovine serum (FBS), L-glutamine and antibiotics penicillin and streptomycin. Cells were cultured at 37 °C in a 5% CO_2_ humidified atmosphere. Culturing medium was replaced every 2–3 days, and cell subculture was conducted once a week by trypsinization.

### 2.2. Permeability Assay

#### 2.2.1. Establishment of a Caco-2 Monolayer

**Figure 1:**
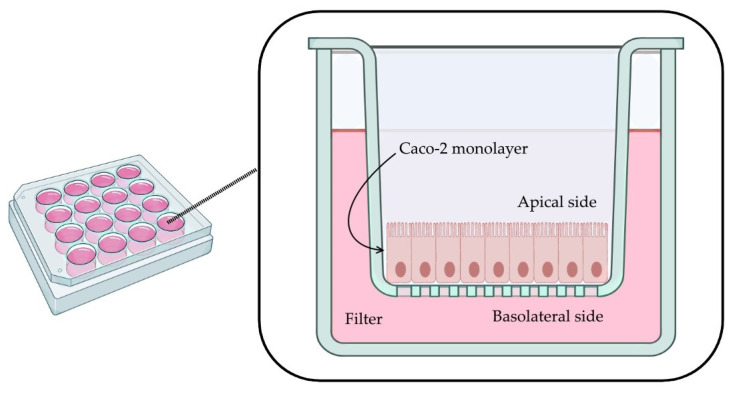
Caco-2 monolayer in permeable filter (illustration created with BioRender [15] for this project).

A monolayer of Caco-2 cells was established in a 12-well plate. Each well contains a permeable filter insert, a transparent collagen-treated (equimolar mixture of types I and III collagen) polytetrafluoroethylene (PTFE) membrane, 12 mm in diameter with a 0.4 µm pore size (Corning Transwell^®^-COL collagen-coated membrane inserts, Corning Inc, Corning, NY, USA, Cat. No. 3493) (Figure 1).

Firstly, filters were pre-wet with 0.1 mL of culture medium for 2 min. Cells were seeded on the apical side (0.5 mL of cells per well, cell density of 4.0 × 10^5^ cells/mL). The basolateral compartment was filled with 1.5 mL of culture medium, and plates were incubated for 6 h (5% CO_2_ humidified atmosphere at 37 °C). Then, to remove nonadherent cells and reduce the risk of multilayer formation, the medium on the apical side was removed and replaced with fresh medium. Cells were maintained for 29 days, replacing the culture medium from both compartments every other day (aspiration from the basal chamber first, followed by careful aspiration from the apical compartment and replacement with the same volume of fresh medium first in the apical compartment and finally in the basal compartment).

#### 2.2.2. Transport across Caco-2 Monolayer Assay

All the solutions used were pre-warmed to 37 °C. Culture medium was replaced with fresh medium 24 h prior to the beginning of the experiment. Then, the culture medium was removed from both compartments, and the apical compartment was carefully washed and filled with 0.5 mL of Hank’s balanced salt solution (HBSS, pH 7.4). The basolateral compartment was also filled with 1.5 mL of HBSS, and the plates were incubated for 17 min at 37 °C under gentle shaking (190 rpm, GFL^®^ Orbital Shaker 3015).

Gemcitabine (purchased from Sigma-Aldrich as gemcitabine hydrochloride, G6423, Algés, Portugal) was prepared in HBBS and added to the apical compartment (final concentration of 60 µM). Throughout this assay, the final volume was 0.4 mL in the apical compartment and 1.2 mL in the basolateral chamber. At t = 0 min, 0.45 mL of the donor solution was added to the apical compartment, and a sample of 0.05 mL was immediately taken. Plates were incubated (lid covered) at 37 °C under gentle shaking (190 rpm). Every 30 min for the next 2 h, a sample of 0.6 mL was taken from the basolateral compartment and replaced with the same volume of HBSS. After 120 min, a sample of 0.05 mL was taken from the apical side. Results are expressed as mean SEM (*n* = 4).

#### 2.2.3. HPLC Quantification

The concentration of gemcitabine in the basolateral and apical compartments was determined by high-performance liquid chromatography (HPLC) (VWR International LCC, LaChrom Ultra, Alfragide, Portugal). Elution was performed with a variable gradient of acetonitrile (ACN) in water containing 0.05% trifluoroacetic acid (TFA) at 0.7 mL/min flow and detection at 243 nm. All chemicals were either analytical or HPLC grade.

### 2.3. PBPK Modeling

GastroPlus™ software version 9.5 (Simulations Plus Inc., Lancaster, CA, USA) was used for absorption modeling and simulation (PBPK modeling), prediction of PK parameters and generation of simulated human plasma concentration profiles. The absorption of oral formulations from the GI tract was modeled by the advanced compartmental absorption and transit (ACAT™) model implemented in GastroPlus™. All simulations were modeled with a compartmental model considering a fasted state. Gemcitabine clearance was inputted as 120 L/h (according to this drug’s FDA label and information deposited on DrugBank) [7,16,17], and the simulation time was set to 24 h for all conditions. The intravenous administration was set as a 30 min infusion of 1800 mg of gemcitabine (standard treatment is 1000 mg/m^2^). For the oral route of administration, an immediate-release tablet was selected, and different treatment regimens were studied: a tablet of 1000 mg once, twice and three times a day and a tablet of 1500 mg twice and three times a day. These dosages were selected as an approximation to the standard treatments. A dose volume of 250 mL was set for all simulations.

## 3. Results and Discussion

### 3.1. Gemcitabine Permeability

**Figure 2:**
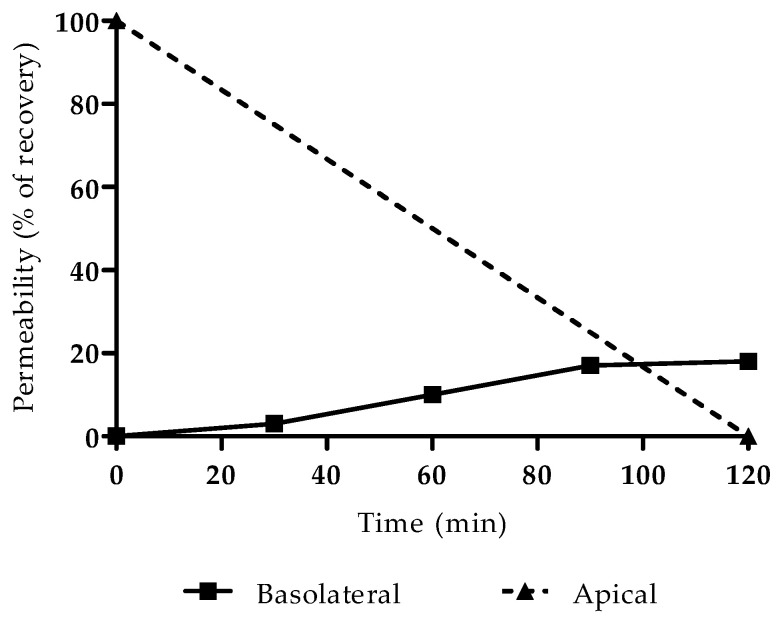
Permeability of gemcitabine, expressed as the percentage recovered in the basolateral and apical compartments.

The successful formation of a monolayer of Caco-2 cells was verified by observation under an optical microscope, with no detection of anomalies or areas without cells, a reliable indicator for proceeding with the permeability study. The integrity of the cells was not compromised by the concentration of gemcitabine used in the permeability assay (60 µM), since the IC50 of this drug is far greater (approximately 50 mM, as determined experimentally via MTT assay and previously reported by Lim et al. [18]). Results are presented in Figure 2 as a percentage of recovery from the basolateral compartment.

The apparent permeability was then calculated as 5.8 × 10^−6^ cm/s using Equation (1),

(1) $$P_{app}=\left(\frac{dQ/dt}{C_{0}\times A}\right)$$

where *dQ/dt* is the amount of compound in the basolateral compartment as a function of time, *C*_0_ is the initial concentration in the donor (apical) compartment and *A* is the area of the transwell filter (cm^2^). This value was inputted in GastroPlus™ and used in all simulations.

### 3.2. PBPK Modeling

Some properties of gemcitabine predicted by GastroPlus™ and parameters used in the simulations are presented in Table 1. The PK parameters predicted from the simulations carried out in this study are presented in Table 2.

In cancer therapy, there are several drugs that are given orally, with dosages of 1000 mg and 1500 mg for the active ingredient. These values were used by us in the simulations to ensure that we had a high dose of the drug for assessing the accumulation and clearance factor when compared to the IV form of gemcitabine administration (Table 2). It is possible to derive a rough estimate of the dose starting from the IV dose (reference) and dividing by the F value of the drug.

**Figure 3:**
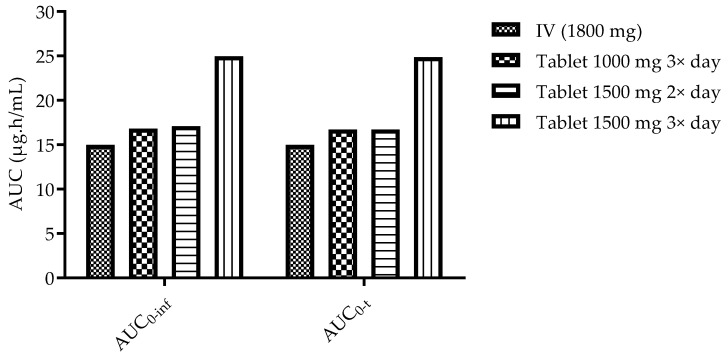
Comparison of AUC between IV infusion (1800 mg), 1000 tablet 3× a day and 1500 mg tablet 2× and 3× a day.

The simulation of the standard treatment via IV infusion provided a prediction of PK parameters approximate to values calculated in studies with cancer patients [19,20,21]. The maximum concentration was estimated as 13.132 mg/L, reached at the end of the infusion (30 min). The C_max_ values predicted for all studied regimens of oral administration were always lower compared to the IV infusion (highest C_max_ predicted was 2.506 mg/L after a tablet of 1500 mg three times a day). However, despite reaching lower gemcitabine concentrations in the plasma, the estimated AUC_0-inf_ was higher for three of the studied oral regimens following tablet administration (16.807 µg·h/mL for 1000 mg tablet 3×/day, 17.095 µg·h/mL for 1500 mg tablet 2× and 24.965 µg·h/mL for 1500 mg tablet 3×/day) compared to the IV administration (14.99 µg·h/mL). This is plausible, since the total daily dose administered is higher. A comparison is depicted and highlighted in Figure 3.

**Figure 4:**
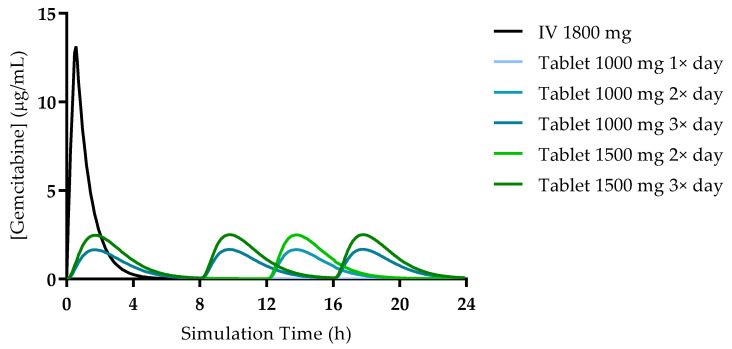
Plasma concentration—time profiles for gemcitabine following 30 min IV infusion and different oral (tablet) treatment regimens.

**Figure 5:**
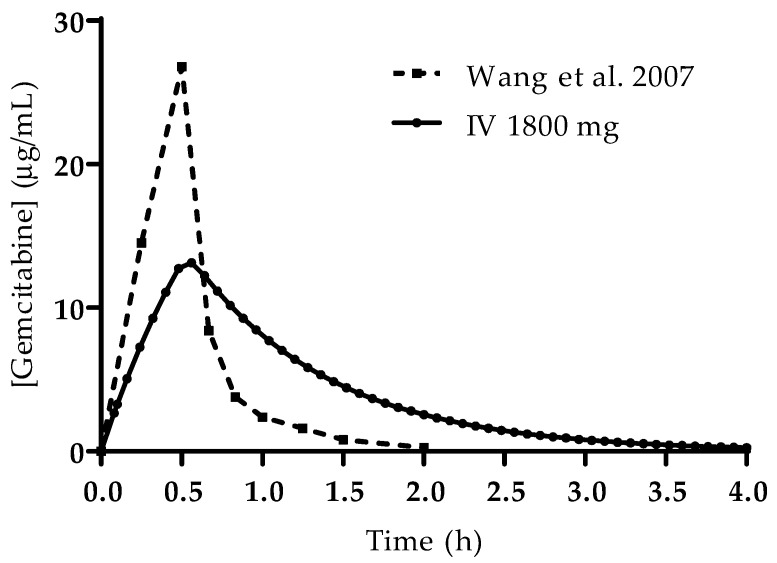
Comparison of the plasma concentration–time profiles from Wang et al. [19] (1200 mg/m^2^ by 30 min IV infusion) and the profile acquired in the simulation carried out in this study (1800 mg by 30 min infusion).

**Figure 6:**
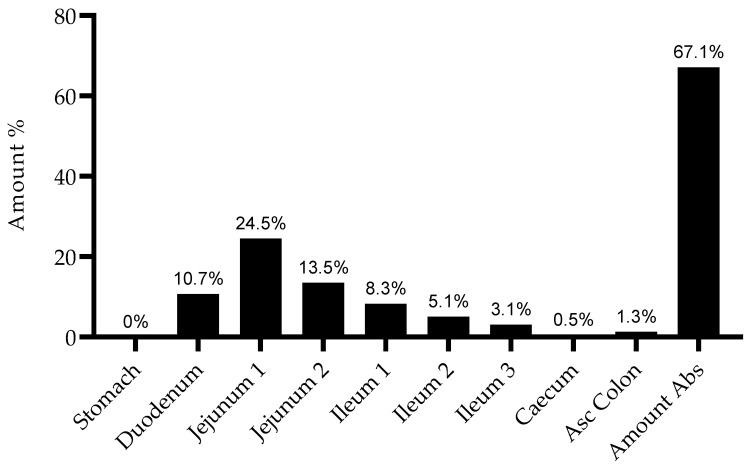
Simulated distribution of gemcitabine absorbed by different compartments after administration of a 1000 mg tablet three times a day.

The simulated plasma concentration–time profiles for all studied conditions are presented in Figure 4. The profile simulated for the IV administration is also consistent with reports from the literature and studies in human patients. Wang et al. studied the PK of gemcitabine in patients with non-small-cell lung cancer (NSCLC) and presented the plasma concentration–time profile for six patients receiving 1200 mg/m^2^ of gemcitabine as a 30 min infusion [19]. A comparison of the profile reported by this group of researchers and the one predicted here is presented in Figure 5. Additionally, it is important to note that there is no drug accumulation in the plasma, even with multiple doses being administered throughout the day and the combined total dose of gemcitabine in some of the studied regimens being higher than 1800 mg (2000 mg, 3000 mg and 4500 mg). The regional absorption of gemcitabine was also analyzed and is presented in Figure 6. These results show that there is no significant difference in the distribution of absorbed gemcitabine between different regimens of oral administration.

## 4. Discussing the Limitations of the Present Study

Despite the convenience of the oral route of administration, there are many aspects to take into consideration regarding this route. As mentioned in the Introduction (Section 1), gemcitabine is significantly metabolized by CDA, present in high levels in the plasma and liver, and is rapidly cleared from the body upon its enzymatic conversion [22,23]. Other enzymes will further contribute to the metabolic transformation and degradation of gemcitabine, including nucleosidase enzymes in the intestinal lumen. Additionally, gemcitabine’s entry into cells is transporter dependent. Not only are equilibrative and concentrative nucleoside transporters required for the perfusion into tissues, gemcitabine will also be a substrate for intestinal nucleoside transporters [24,25].

Regarding another considerable factor, gemcitabine’s permeability has been previously assessed in in vitro and in vivo mouse models [24] and has been evaluated here in a Caco-2 monolayer of cells to mimic the intestinal epithelium. Although Caco-2 permeability values do not directly translate in vivo permeability, in this project, this was used to help parametrize the PBPK models.

The oral administration of gemcitabine was previously evaluated in patients with refractory tumors by Veltkamp et al., in much lower doses than the ones evaluated here, ranging from 2 to 20 mg [26]. However, in this preliminary work, the chosen doses were closer to the ones intravenously administered in conventional therapy, to assess the exposure via the oral route of administration.

In sum, this work intended to analyze the pharmacokinetic viability of the oral route of administration for gemcitabine. Taking the results observed, progressing with this study will imply acquiring more data relating to the metabolism, transport and, later, the pharmacodynamic aspects of this drug.

## 5. Conclusions

Given the major drawbacks of chemotherapy, we were interested in studying an alternative to the standard treatment regimen for gemcitabine that includes IV infusions. Here, we developed several PBPK models and studied the PK of this drug after different regimens of oral administration via tablet, an easier and more comfortable route of administration for patients. The results from our simulations showed that despite the estimated C_max_ being lower for all regimens of oral administration, the predicted AUC is higher for three of the studies conditions (1000 mg tablet 3×/day, 1500 mg tablet 2×–3×/day) compared to the IV administration. This can indicate an enhanced exposure to this drug, retaining the therapeutic effect despite the lower concentration in the plasma. Furthermore, there was no drug accumulation even with multiple doses a day and a total dose higher than 1800 mg (maximum daily dose of 4500 mg). Taking together the results from the present study, we believe that oral administration of gemcitabine is a promising and viable alternative to the current standard IV regimen, since it can allow high drug exposure, and that other therapeutic options are worthy of further study. It would also be important to note that the distribution remains the same because it can be influenced by the physicochemical properties of gemcitabine and not by the route of absorption. In addition, the absence of accumulation may also be expected as the daily dose administered is not significantly different from that of the IV infusion.

This study involving physiologically based pharmacokinetic modeling combines the system-dependent physiological, anatomical and biochemical properties, specific properties of gemcitabine, as well as the formulation parameters, providing an approach to predict the plasma concentration–time profiles. We believe these can be important to support decision making throughout the drug research and development. Additionally, it is possible to use this information to determine the best dosing regimen for an effective and safe concentration, using the patient covariate values. This can be an example of personalized medicine based on potential new formulations for oral administration of gemcitabine, as well as nanotechnology-based drug delivery systems.

Switching from IV to the oral route (and vice versa) may be based not only on a comparison of C_p_-time profiles but also on PBPK modeling. Therefore, joint PBPK simulations can be performed to examine the simulated PD effect (which may serve as a surrogate for the clinical effect). A robust gemcitabine PBPK model has not been developed yet. PBPK model evaluation can be performed using several methods. Model predictions of plasma concentration–time profiles can be graphically compared to observed profiles from the respective clinical studies. Subsequently, predicted plasma concentrations from all studies will be plotted against their corresponding observed values in goodness-of-fit plots.

## Figures and Tables

**Table 1:** Structure, parameters and gemcitabine properties predicted by GastroPlus™ used in the simulations.

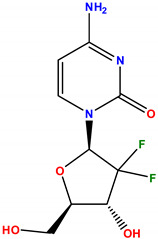		
Parameter	Value	Source
Molecular weight	263.2	GastroPlus™
logP (neutral)	−1.32	
Solubility	5.01 mg/mL (at pH 7.92)	
Mean precipitation time	900 s	
Drug particle density	1.2 g/mL	
Diffusion coefficient	0.93 × 10^5^ cm^2^/s	
Blood/plasma concentration ratio	1.12	
Human jejunal permeability	0.59 × 10^−4^ cm/s	GastroPlus™ and experimental determination
F_up_	84.60%	GastroPlus™ (>90% in [7,16])
V_c_	1.45 L/kg	GastroPlus™ (1.3 L/kg in [7,16])
T_1/2_	0.59 h	GastroPlus™ (0.7 to 1.57 h in [7,16])

**Table 2:** Predicted pharmacokinetic properties of gemcitabine determined with GastroPlus™ for different treatment conditions.

Posology			Fa (%) ^1^	FDp (%) ^2^	F (%) ^3^	C_max_ (mg/L) ^4^	T_max_ (h) ^5^	AUC_0-inf_ (µg·h/mL) ^6^	AUC_0-t_ (µg·h/mL) ^7^	C_max liver_ (mg/L) ^8^
Admin. Route	Dose (mg)	Interval								
IV	1800	---	99.929	99.929	99.929	13.132	0.50	14.990	14.989	12.5610
Tablet (Immediate release)	1000	24 h (1×/day)	68.026	68.013	68.013	1.656	1.68	5.732	5.663	3.9916
		12 h (2×/day)	67.411	67.387	67.387	1.668	13.68	11.458	11.214	4.0185
		8 h (3×/day)	67.051	67.020	67.020	1.684	17.68	16.807	16.711	4.0517
	1500	12 h (2×/day)	66.945	66.925	66.925	2.493	13.68	17.095	16.711	6.0148
		8 h (3×/day)	66.460	66.433	66.433	2.506	17.68	24.965	24.855	6.0435

^1^ Fraction absorbed as a percent of the dose (crossing the lumen and entering enterocytes). ^2^ Percent of the dose that has reached the portal vein. ^3^ Bioavailability. ^4^ Maximum plasma concentration reached in the central compartment, in mg/L. ^5^ Time to reach maximum plasma concentration, in hours. ^6^ Area under the plasma concentration–time curve, in µg·h/mL, extrapolated to infinity. ^7^ Area under the plasma concentration–time curve, in µg·h/mL, for the time of the simulation. ^8^ Maximum concentration reached in the liver, in mg/L.

## Data Availability

Not applicable.
